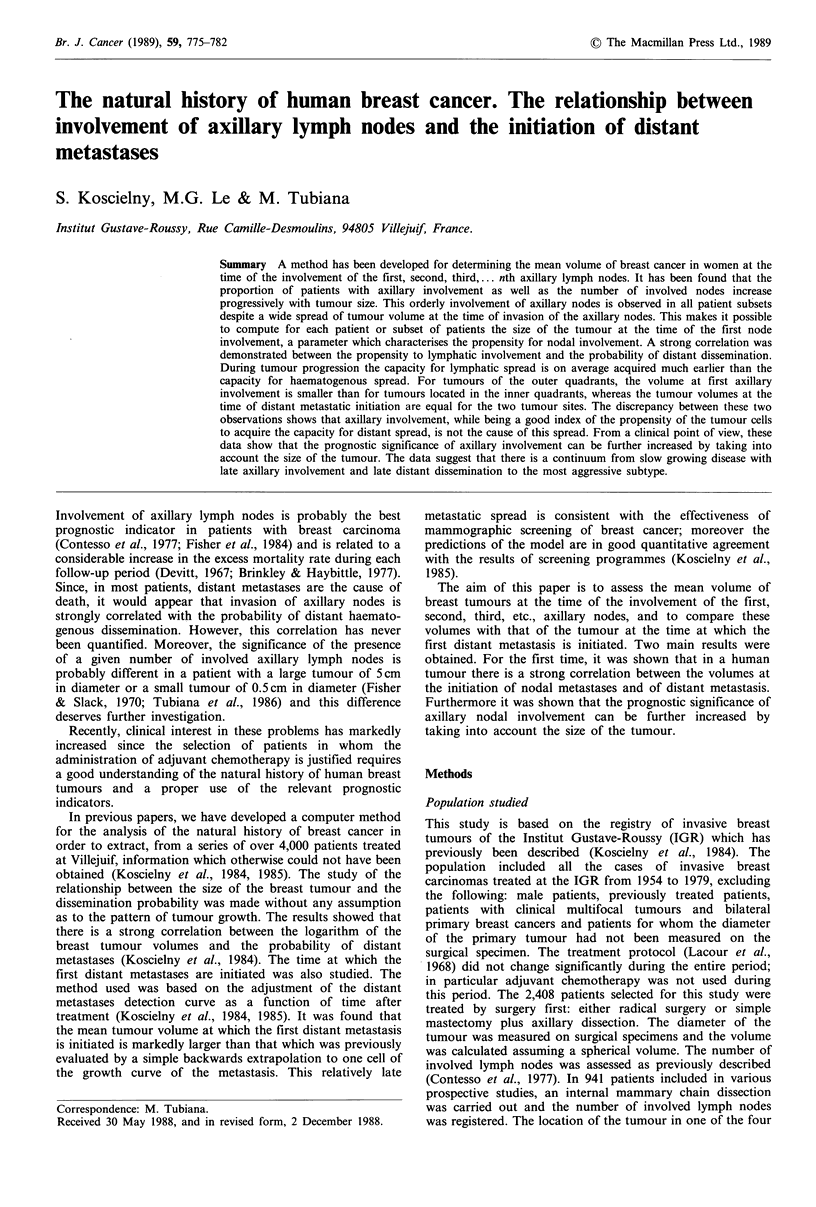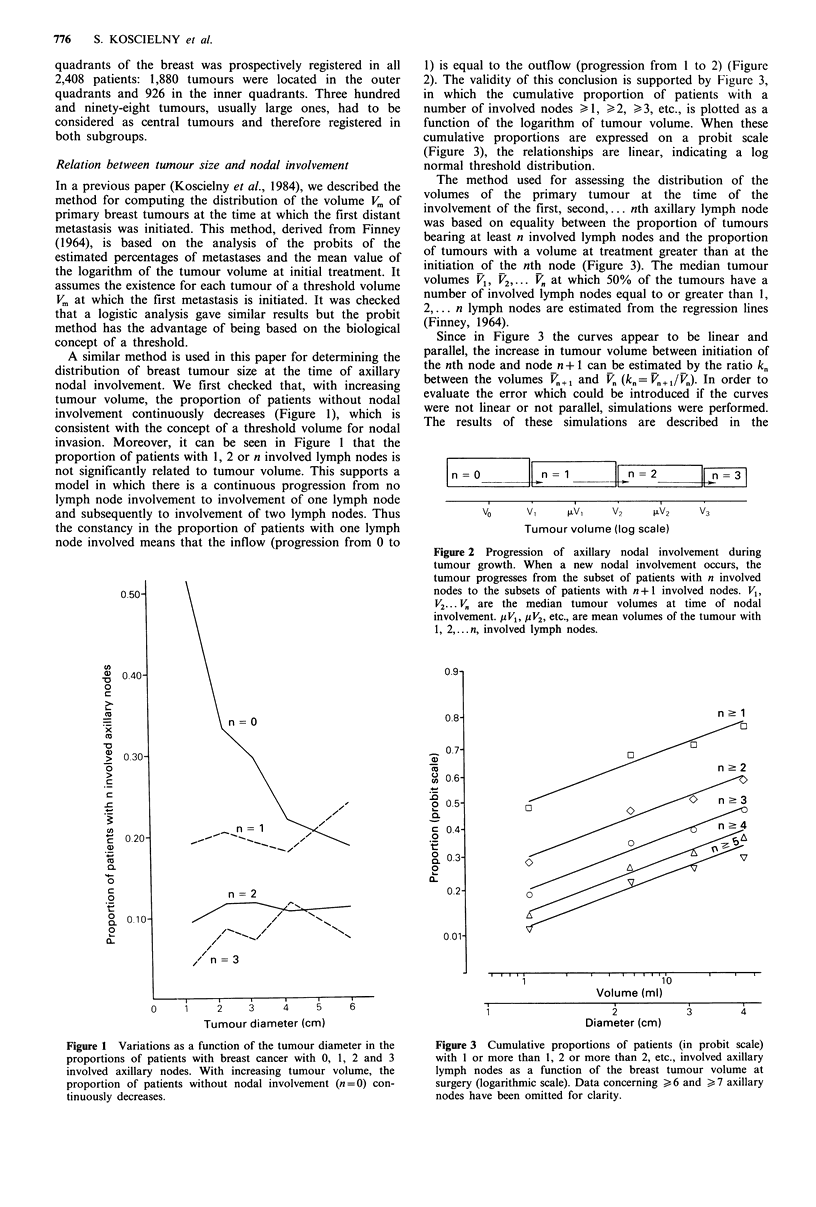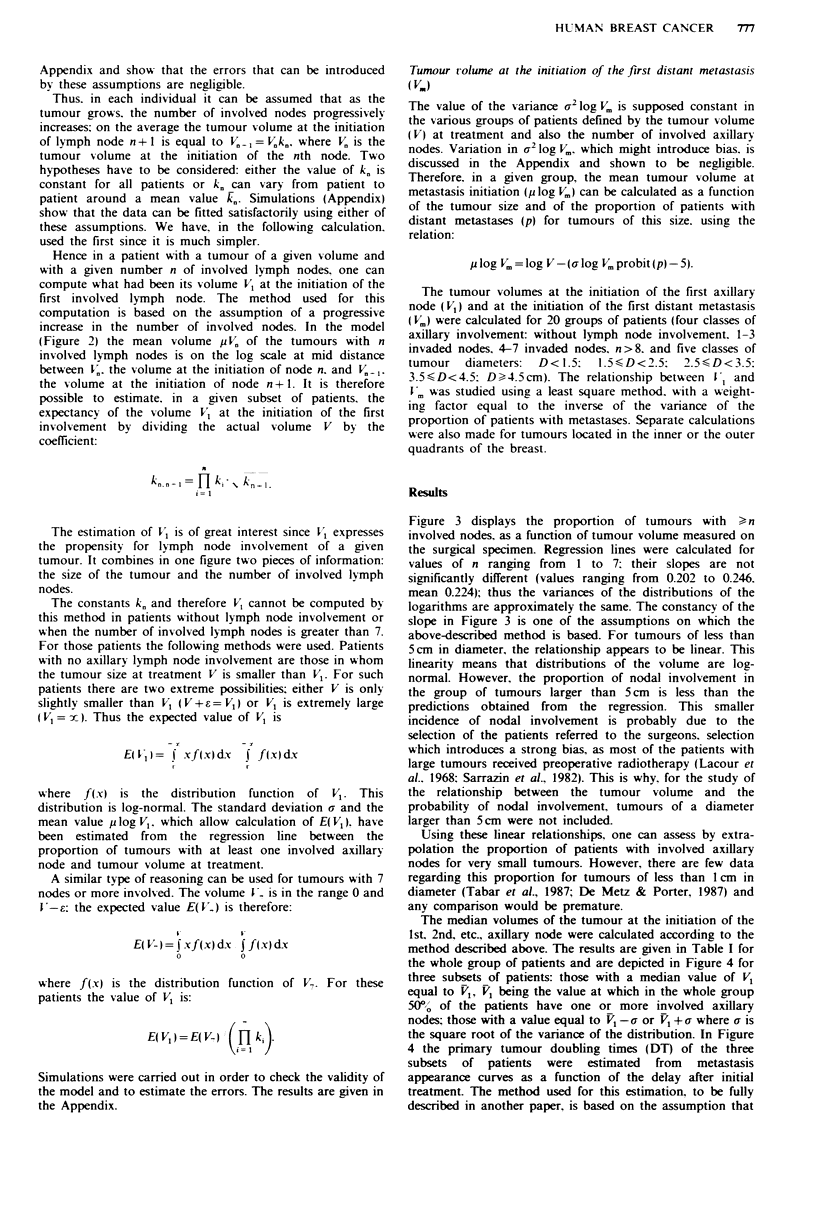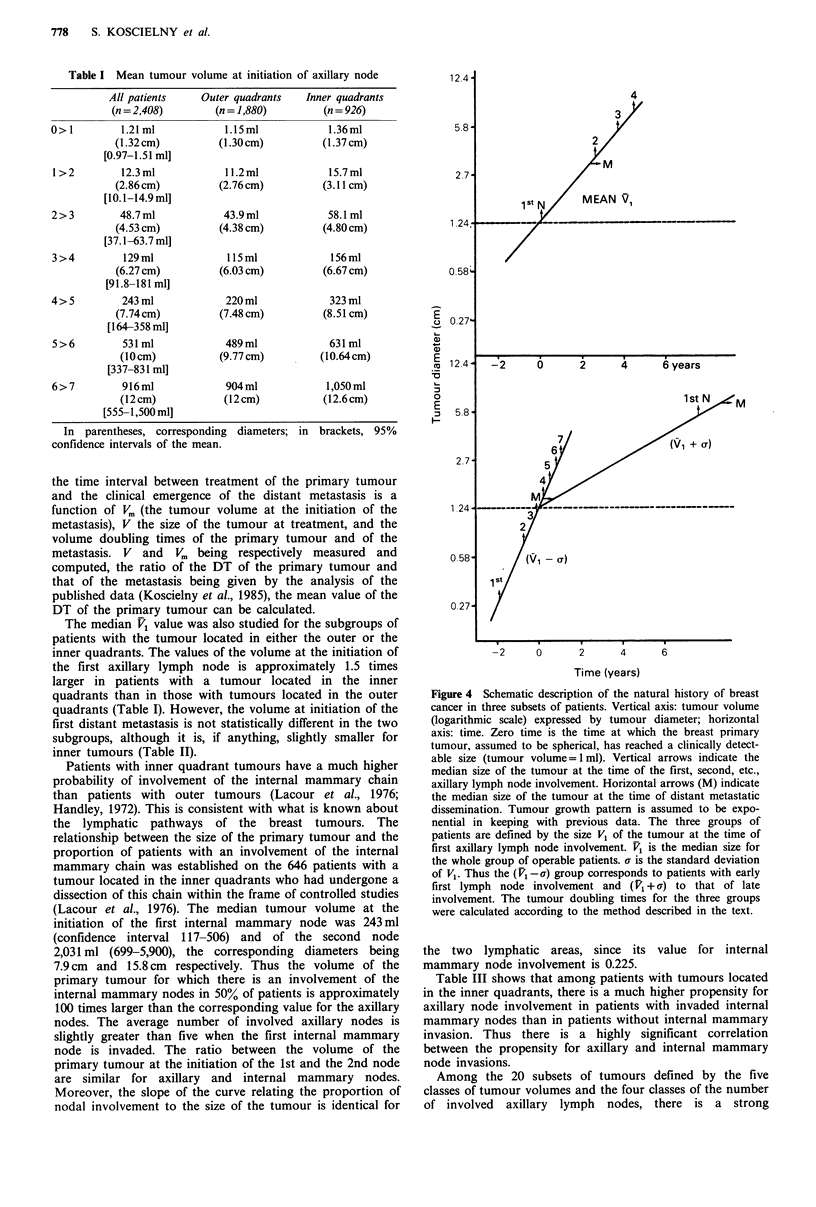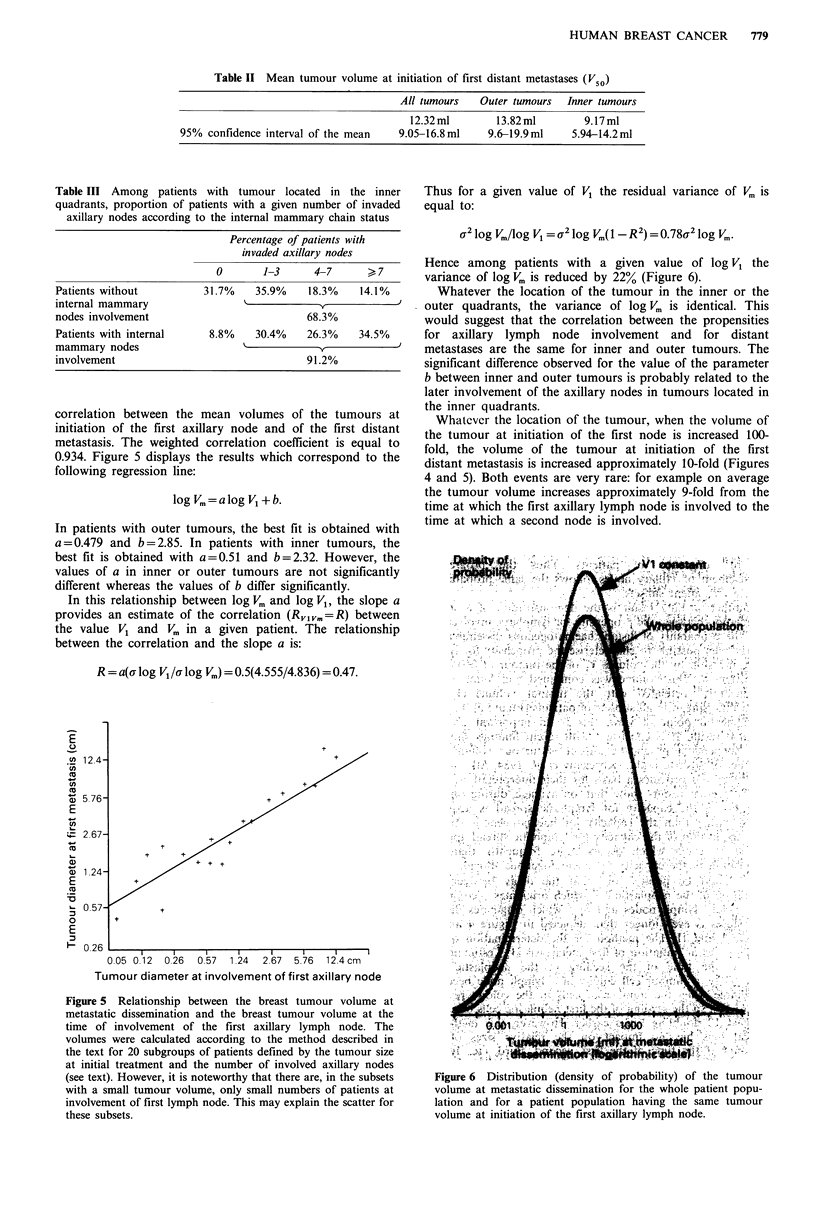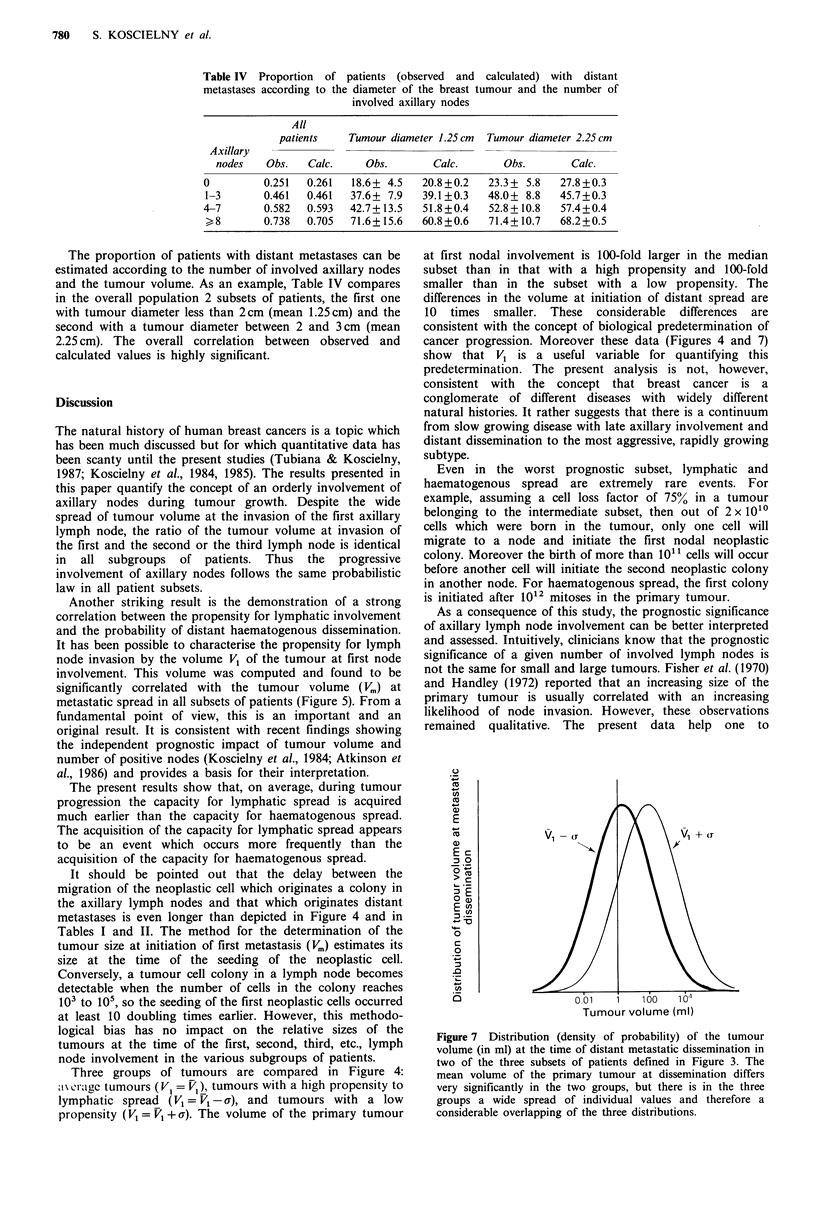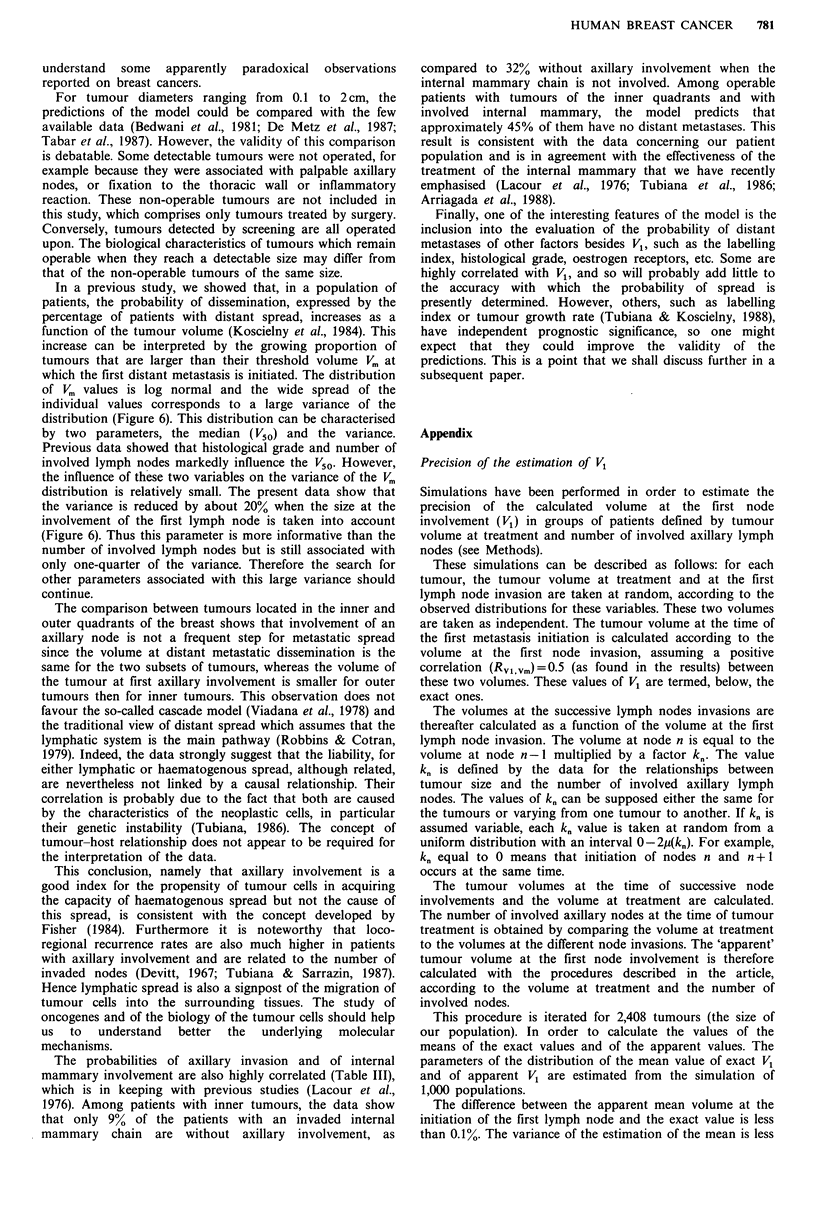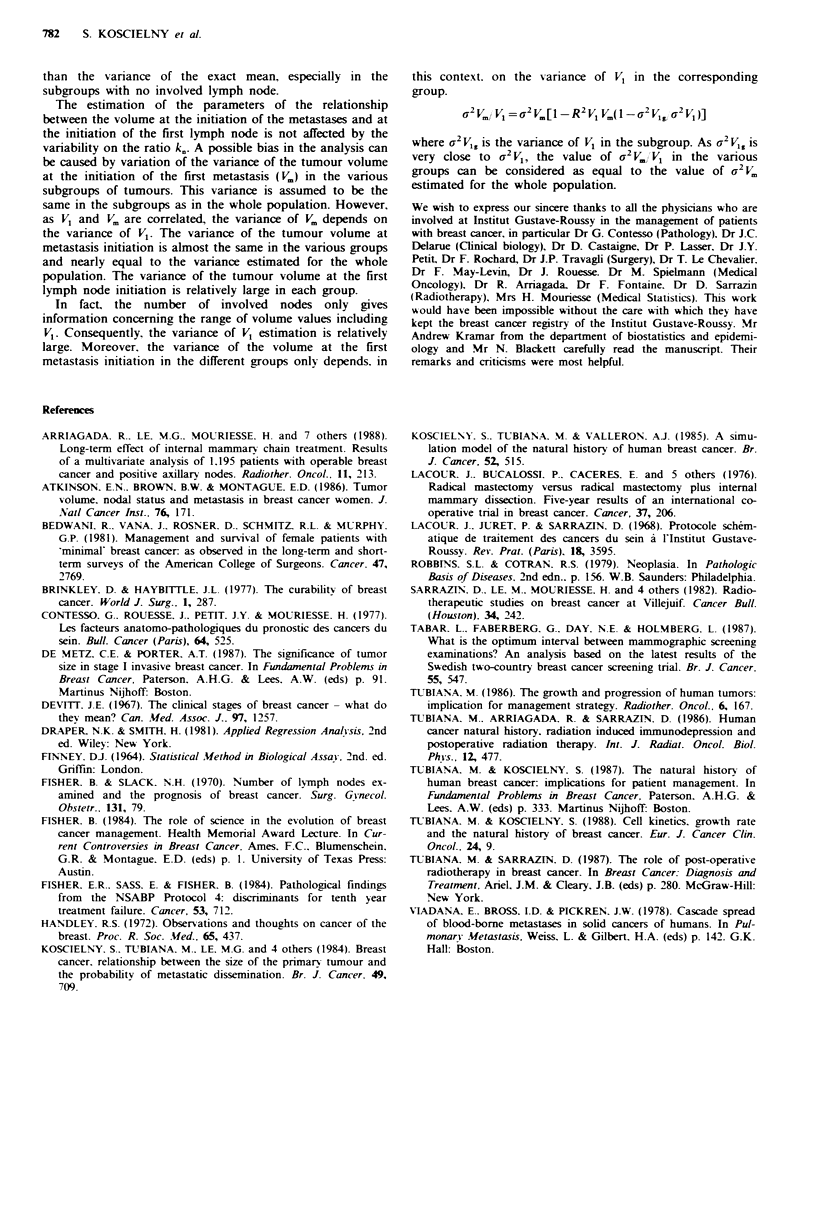# The natural history of human breast cancer. The relationship between involvement of axillary lymph nodes and the initiation of distant metastases.

**DOI:** 10.1038/bjc.1989.162

**Published:** 1989-05

**Authors:** S. Koscielny, M. G. Le, M. Tubiana

**Affiliations:** Institut Gustave-Roussy, Villejuif, France.

## Abstract

A method has been developed for determining the mean volume of breast cancer in women at the time of the involvement of the first, second, third,... nth axillary lymph nodes. It has been found that the proportion of patients with axillary involvement as well as the number of involved nodes increase progressively with tumour size. This orderly involvement of axillary nodes is observed in all patient subsets despite a wide spread of tumour volume at the time of invasion of the axillary nodes. This makes it possible to compute for each patient or subset of patients the size of the tumour at the time of the first node involvement, a parameter which characterises the propensity for nodal involvement. A strong correlation was demonstrated between the propensity to lymphatic involvement and the probability of distant dissemination. During tumour progression the capacity for lymphatic spread is on average acquired much earlier than the capacity for haematogenous spread. For tumours of the outer quadrants, the volume at first axillary involvement is smaller than for tumours located in the inner quadrants, whereas the tumour volumes at the time of distant metastatic initiation are equal for the two tumour sites. The discrepancy between these two observations shows that axillary involvement, while being a good index of the propensity of the tumour cells to acquire the capacity for distant spread, is not the cause of this spread. From a clinical point of view, these data show that the prognostic significance of axillary involvement can be further increased by taking into account the size of the tumour. The data suggest that there is a continuum from slow growing disease with late axillary involvement and late distant dissemination to the most aggressive subtype.


Br. J. Cancer (1989), 59, 775–782 © The Macmillan Press Ltd., 1989

### The natural history of human breast cancer. The relationship between involvement of axillary lymph nodes and the initiation of distant metastases

S. Koscielny, M.G. Le & M. Tubiana

*Institut Gustave-Roussy, Rue Camille-Desmoulins, 94805 Villejuif, France.*

**Summary** A method has been developed for determining the mean volume of breast cancer in women at the time of the involvement of the first, second, third,... *n*th axillary lymph nodes. It has been found that the proportion of patients with axillary involvement as well as the number of involved nodes increase progressively with tumour size. This orderly involvement of axillary nodes is observed in all patient subsets despite a wide spread of tumour volume at the time of invasion of the axillary nodes. This makes it possible to compute for each patient or subset of patients the size of the tumour at the time of the first node involvement, a parameter which characterises the propensity for nodal involvement. A strong correlation was demonstrated between the propensity to lymphatic involvement and the probability of distant dissemination. During tumour progression the capacity for lymphatic spread is on average acquired much earlier than the capacity for haematogenous spread. For tumours of the outer quadrants, the volume at first axillary involvement is smaller than for tumours located in the inner quadrants, whereas the tumour volumes at the time of distant metastatic initiation are equal for the two tumour sites. The discrepancy between these two observations shows that axillary involvement, while being a good index of the propensity of the tumour cells to acquire the capacity for distant spread, is not the cause of this spread. From a clinical point of view, these data show that the prognostic significance of axillary involvement can be further increased by taking into account the size of the tumour. The data suggest that there is a continuum from slow growing disease with late axillary involvement and late distant dissemination to the most aggressive subtype.

Involvement of axillary lymph nodes is probably the best prognostic indicator in patients with breast carcinoma (Contesso *et al.*, 1977; Fisher *et al.*, 1984) and is related to a considerable increase in the excess mortality rate during each follow-up period (Devitt, 1967; Brinkley & Haybittle, 1977). Since, in most patients, distant metastases are the cause of death, it would appear that invasion of axillary nodes is strongly correlated with the probability of distant haematogenous dissemination. However, this correlation has never been quantified. Moreover, the significance of the presence of a given number of involved axillary lymph nodes is probably different in a patient with a large tumour of 5 cm in diameter or a small tumour of 0.5 cm in diameter (Fisher & Slack, 1970; Tubiana *et al.*, 1986) and this difference deserves further investigation.

Recently, clinical interest in these problems has markedly increased since the selection of patients in whom the administration of adjuvant chemotherapy is justified requires a good understanding of the natural history of human breast tumours and a proper use of the relevant prognostic indicators.

In previous papers, we have developed a computer method for the analysis of the natural history of breast cancer in order to extract, from a series of over 4,000 patients treated at Villejuif, information which otherwise could not have been obtained (Koscielny *et al.*, 1984, 1985). The study of the relationship between the size of the breast tumour and the dissemination probability was made without any assumption as to the pattern of tumour growth. The results showed that there is a strong correlation between the logarithm of the breast tumour volumes and the probability of distant metastases (Koscielny *et al.*, 1984). The time at which the first distant metastases are initiated was also studied. The method used was based on the adjustment of the distant metastases detection curve as a function of time after treatment (Koscielny *et al.*, 1984, 1985). It was found that the mean tumour volume at which the first distant metastasis is initiated is markedly larger than that which was previously evaluated by a simple backwards extrapolation to one cell of the growth curve of the metastasis. This relatively late metastatic spread is consistent with the effectiveness of mammographic screening of breast cancer; moreover the predictions of the model are in good quantitative agreement with the results of screening programmes (Koscielny *et al.*, 1985).

The aim of this paper is to assess the mean volume of breast tumours at the time of the involvement of the first, second, third, etc., axillary nodes, and to compare these volumes with that of the tumour at the time at which the first distant metastasis is initiated. Two main results were obtained. For the first time, it was shown that in a human tumour there is a strong correlation between the volumes at the initiation of nodal metastases and of distant metastasis. Furthermore it was shown that the prognostic significance of axillary nodal involvement can be further increased by taking into account the size of the tumour.

### Methods

### *Population studied*

This study is based on the registry of invasive breast tumours of the Institut Gustave-Roussy (IGR) which has previously been described (Koscielny *et al.*, 1984). The population included all the cases of invasive breast carcinomas treated at the IGR from 1954 to 1979, excluding the following: male patients, previously treated patients, patients with clinical multifocal tumours and bilateral primary breast cancers and patients for whom the diameter of the primary tumour had not been measured on the surgical specimen. The treatment protocol (Lacour *et al.*, 1968) did not change significantly during the entire period; in particular adjuvant chemotherapy was not used during this period. The 2,408 patients selected for this study were treated by surgery first: either radical surgery or simple mastectomy plus axillary dissection. The diameter of the tumour was measured on surgical specimens and the volume was calculated assuming a spherical volume. The number of involved lymph nodes was assessed as previously described (Contesso *et al.*, 1977). In 941 patients included in various prospective studies, an internal mammary chain dissection was carried out and the number of involved lymph nodes was registered. The location of the tumour in one of the four

Correspondence: M. Tubiana.
Received 30 May 1988, and in revised form, 2 December 1988.

quadrants of the breast was prospectively registered in all 2,408 patients: 1,880 tumours were located in the outer quadrants and 926 in the inner quadrants. Three hundred and ninety-eight tumours, usually large ones, had to be considered as central tumours and therefore registered in both subgroups.

*Relation between tumour size and nodal involvement*

In a previous paper (Koscielny *et al.*, 1984), we described the method for computing the distribution of the volume $V_m$ of primary breast tumours at the time at which the first distant metastasis was initiated. This method, derived from Finney (1964), is based on the analysis of the probits of the estimated percentages of metastases and the mean value of the logarithm of the tumour volume at initial treatment. It assumes the existence for each tumour of a threshold volume $V_m$ at which the first metastasis is initiated. It was checked that a logistic analysis gave similar results but the probit method has the advantage of being based on the biological concept of a threshold.

A similar method is used in this paper for determining the distribution of breast tumour size at the time of axillary nodal involvement. We first checked that, with increasing tumour volume, the proportion of patients without nodal involvement continuously decreases (Figure 1), which is consistent with the concept of a threshold volume for nodal invasion. Moreover, it can be seen in Figure 1 that the proportion of patients with 1, 2 or $n$ involved lymph nodes is not significantly related to tumour volume. This supports a model in which there is a continuous progression from no lymph node involvement to involvement of one lymph node and subsequently to involvement of two lymph nodes. Thus the constancy in the proportion of patients with one lymph node involved means that the inflow (progression from 0 to 1) is equal to the outflow (progression from 1 to 2) (Figure 2). The validity of this conclusion is supported by Figure 3, in which the cumulative proportion of patients with a number of involved nodes $\geqslant 1$, $\geqslant 2$, $\geqslant 3$, etc., is plotted as a function of the logarithm of tumour volume. When these cumulative proportions are expressed on a probit scale (Figure 3), the relationships are linear, indicating a log normal threshold distribution.

The method used for assessing the distribution of the volumes of the primary tumour at the time of the involvement of the first, second,... $n$th axillary lymph node was based on equality between the proportion of tumours bearing at least $n$ involved lymph nodes and the proportion of tumours with a volume at treatment greater than at the initiation of the $n$th node (Figure 3). The median tumour volumes $\bar{V}_1$, $\bar{V}_2$,... $\bar{V}_n$ at which 50% of the tumours have a number of involved lymph nodes equal to or greater than 1, 2,... $n$ lymph nodes are estimated from the regression lines (Finney, 1964).

Since in Figure 3 the curves appear to be linear and parallel, the increase in tumour volume between initiation of the $n$th node and node $n+1$ can be estimated by the ratio $k_n$ between the volumes $\bar{V}_{n+1}$ and $\bar{V}_n$ ($k_n = \bar{V}_{n+1}/\bar{V}_n$). In order to evaluate the error which could be introduced if the curves were not linear or not parallel, simulations were performed. The results of these simulations are described in the

**Figure 2** Progression of axillary nodal involvement during tumour growth. When a new nodal involvement occurs, the tumour progresses from the subset of patients with $n$ involved nodes to the subsets of patients with $n+1$ involved nodes. $V_1$, $V_2$... $V_n$ are the median tumour volumes at time of nodal involvement. $\mu V_1$, $\mu V_2$, etc., are mean volumes of the tumour with 1, 2,... $n$, involved lymph nodes.

**Figure 1** Variations as a function of the tumour diameter in the proportions of patients with breast cancer with 0, 1, 2 and 3 involved axillary nodes. With increasing tumour volume, the proportion of patients without nodal involvement ($n=0$) continuously decreases.

**Figure 3** Cumulative proportions of patients (in probit scale) with 1 or more than 1, 2 or more than 2, etc., involved axillary lymph nodes as a function of the breast tumour volume at surgery (logarithmic scale). Data concerning $\geqslant 6$ and $\geqslant 7$ axillary nodes have been omitted for clarity.

Appendix and show that the errors that can be introduced by these assumptions are negligible.

Thus, in each individual it can be assumed that as the tumour grows, the number of involved nodes progressively increases; on the average the tumour volume at the initiation of lymph node $n+1$ is equal to $V_{n+1} = V_n k_n$, where $V_n$ is the tumour volume at the initiation of the $n$th node. Two hypotheses have to be considered: either the value of $k_n$ is constant for all patients or $k_n$ can vary from patient to patient around a mean value $\bar{k}_n$. Simulations (Appendix) show that the data can be fitted satisfactorily using either of these assumptions. We have, in the following calculation, used the first since it is much simpler.

Hence in a patient with a tumour of a given volume and with a given number $n$ of involved lymph nodes, one can compute what had been its volume $V_1$ at the initiation of the first involved lymph node. The method used for this computation is based on the assumption of a progressive increase in the number of involved nodes. In the model (Figure 2) the mean volume $\mu V_n$ of the tumours with $n$ involved lymph nodes is on the log scale at mid distance between $V_n$, the volume at the initiation of node $n$, and $V_{n+1}$, the volume at the initiation of node $n+1$. It is therefore possible to estimate, in a given subset of patients, the expectancy of the volume $V_1$ at the initiation of the first involvement by dividing the actual volume $V$ by the coefficient:

$$k_{n,n-1} = \prod_{i=1}^{n} k_i \cdot \sqrt{k_{n+1}}.$$

The estimation of $V_1$ is of great interest since $V_1$ expresses the propensity for lymph node involvement of a given tumour. It combines in one figure two pieces of information: the size of the tumour and the number of involved lymph nodes.

The constants $k_n$ and therefore $V_1$ cannot be computed by this method in patients without lymph node involvement or when the number of involved lymph nodes is greater than 7. For those patients the following methods were used. Patients with no axillary lymph node involvement are those in whom the tumour size at treatment $V$ is smaller than $V_1$. For such patients there are two extreme possibilities: either $V$ is only slightly smaller than $V_1$ ($V+\varepsilon = V_1$) or $V_1$ is extremely large ($V_1 = \infty$). Thus the expected value of $V_1$ is

$$E(V_1) = \int_V^{\infty} x f(x)\,\mathrm{d}x \Big/ \int_V^{\infty} f(x)\,\mathrm{d}x$$

where $f(x)$ is the distribution function of $V_1$. This distribution is log-normal. The standard deviation $\sigma$ and the mean value $\mu \log V_1$, which allow calculation of $E(V_1)$, have been estimated from the regression line between the proportion of tumours with at least one involved axillary node and tumour volume at treatment.

A similar type of reasoning can be used for tumours with 7 nodes or more involved. The volume $V_7$ is in the range 0 and $V-\varepsilon$; the expected value $E(V_7)$ is therefore:

$$E(V_7) = \int_0^{V} x f(x)\,\mathrm{d}x \Big/ \int_0^{V} f(x)\,\mathrm{d}x$$

where $f(x)$ is the distribution function of $V_7$. For these patients the value of $V_1$ is:

$$E(V_1) = E(V_7) \Big/ \left(\prod_{i=1}^{7} k_i\right).$$

Simulations were carried out in order to check the validity of the model and to estimate the errors. The results are given in the Appendix.

*Tumour volume at the initiation of the first distant metastasis* ($V_m$)

The value of the variance $\sigma^2 \log V_m$ is supposed constant in the various groups of patients defined by the tumour volume ($V$) at treatment and also the number of involved axillary nodes. Variation in $\sigma^2 \log V_m$, which might introduce bias, is discussed in the Appendix and shown to be negligible. Therefore, in a given group, the mean tumour volume at metastasis initiation ($\mu \log V_m$) can be calculated as a function of the tumour size and of the proportion of patients with distant metastases ($p$) for tumours of this size, using the relation:

$$\mu \log V_m = \log V - (\sigma \log V_m \,\mathrm{probit}(p) - 5).$$

The tumour volumes at the initiation of the first axillary node ($V_1$) and at the initiation of the first distant metastasis ($V_m$) were calculated for 20 groups of patients (four classes of axillary involvement: without lymph node involvement, 1–3 invaded nodes, 4–7 invaded nodes, $n>8$, and five classes of tumour diameters: $D<1.5$; $1.5 \leqslant D < 2.5$; $2.5 \leqslant D < 3.5$; $3.5 \leqslant D < 4.5$; $D \geqslant 4.5$ cm). The relationship between $V_1$ and $V_m$ was studied using a least square method, with a weighting factor equal to the inverse of the variance of the proportion of patients with metastases. Separate calculations were also made for tumours located in the inner or the outer quadrants of the breast.

### Results

Figure 3 displays the proportion of tumours with $\geqslant n$ involved nodes, as a function of tumour volume measured on the surgical specimen. Regression lines were calculated for values of $n$ ranging from 1 to 7; their slopes are not significantly different (values ranging from 0.202 to 0.246, mean 0.224); thus the variances of the distributions of the logarithms are approximately the same. The constancy of the slope in Figure 3 is one of the assumptions on which the above-described method is based. For tumours of less than 5 cm in diameter, the relationship appears to be linear. This linearity means that distributions of the volume are log-normal. However, the proportion of nodal involvement in the group of tumours larger than 5 cm is less than the predictions obtained from the regression. This smaller incidence of nodal involvement is probably due to the selection of the patients referred to the surgeons, selection which introduces a strong bias, as most of the patients with large tumours received preoperative radiotherapy (Lacour *et al.*, 1968; Sarrazin *et al.*, 1982). This is why, for the study of the relationship between the tumour volume and the probability of nodal involvement, tumours of a diameter larger than 5 cm were not included.

Using these linear relationships, one can assess by extrapolation the proportion of patients with involved axillary nodes for very small tumours. However, there are few data regarding this proportion for tumours of less than 1 cm in diameter (Tabar *et al.*, 1987; De Metz & Porter, 1987) and any comparison would be premature.

The median volumes of the tumour at the initiation of the 1st, 2nd, etc., axillary node were calculated according to the method described above. The results are given in Table I for the whole group of patients and are depicted in Figure 4 for three subsets of patients: those with a median value of $V_1$ equal to $\bar{V}_1$, $\bar{V}_1$ being the value at which in the whole group 50% of the patients have one or more involved axillary nodes; those with a value equal to $\bar{V}_1 - \sigma$ or $\bar{V}_1 + \sigma$ where $\sigma$ is the square root of the variance of the distribution. In Figure 4 the primary tumour doubling times (DT) of the three subsets of patients were estimated from metastasis appearance curves as a function of the delay after initial treatment. The method used for this estimation, to be fully described in another paper, is based on the assumption that

**Table I** Mean tumour volume at initiation of axillary node

| | *All patients* (*n*=*2,408*) | *Outer quadrants* (*n*=*1,880*) | *Inner quadrants* (*n*=*926*) |
|---|---|---|---|
| 0>1 | 1.21 ml (1.32 cm) [0.97–1.51 ml] | 1.15 ml (1.30 cm) | 1.36 ml (1.37 cm) |
| 1>2 | 12.3 ml (2.86 cm) [10.1–14.9 ml] | 11.2 ml (2.76 cm) | 15.7 ml (3.11 cm) |
| 2>3 | 48.7 ml (4.53 cm) [37.1–63.7 ml] | 43.9 ml (4.38 cm) | 58.1 ml (4.80 cm) |
| 3>4 | 129 ml (6.27 cm) [91.8–181 ml] | 115 ml (6.03 cm) | 156 ml (6.67 cm) |
| 4>5 | 243 ml (7.74 cm) [164–358 ml] | 220 ml (7.48 cm) | 323 ml (8.51 cm) |
| 5>6 | 531 ml (10 cm) [337–831 ml] | 489 ml (9.77 cm) | 631 ml (10.64 cm) |
| 6>7 | 916 ml (12 cm) [555–1,500 ml] | 904 ml (12 cm) | 1,050 ml (12.6 cm) |

In parentheses, corresponding diameters; in brackets, 95% confidence intervals of the mean.

the time interval between treatment of the primary tumour and the clinical emergence of the distant metastasis is a function of $V_m$ (the tumour volume at the initiation of the metastasis), $V$ the size of the tumour at treatment, and the volume doubling times of the primary tumour and of the metastasis. $V$ and $V_m$ being respectively measured and computed, the ratio of the DT of the primary tumour and that of the metastasis being given by the analysis of the published data (Koscielny *et al.*, 1985), the mean value of the DT of the primary tumour can be calculated.

The median $\bar{V}_1$ value was also studied for the subgroups of patients with the tumour located in either the outer or the inner quadrants. The values of the volume at the initiation of the first axillary lymph node is approximately 1.5 times larger in patients with a tumour located in the inner quadrants than in those with tumours located in the outer quadrants (Table I). However, the volume at initiation of the first distant metastasis is not statistically different in the two subgroups, although it is, if anything, slightly smaller for inner tumours (Table II).

Patients with inner quadrant tumours have a much higher probability of involvement of the internal mammary chain than patients with outer tumours (Lacour *et al.*, 1976; Handley, 1972). This is consistent with what is known about the lymphatic pathways of the breast tumours. The relationship between the size of the primary tumour and the proportion of patients with an involvement of the internal mammary chain was established on the 646 patients with a tumour located in the inner quadrants who had undergone a dissection of this chain within the frame of controlled studies (Lacour *et al.*, 1976). The median tumour volume at the initiation of the first internal mammary node was 243 ml (confidence interval 117–506) and of the second node 2,031 ml (699–5,900), the corresponding diameters being 7.9 cm and 15.8 cm respectively. Thus the volume of the primary tumour for which there is an involvement of the internal mammary nodes in 50% of patients is approximately 100 times larger than the corresponding value for the axillary nodes. The average number of involved axillary nodes is slightly greater than five when the first internal mammary node is invaded. The ratio between the volume of the primary tumour at the initiation of the 1st and the 2nd node are similar for axillary and internal mammary nodes. Moreover, the slope of the curve relating the proportion of nodal involvement to the size of the tumour is identical for

**Figure 4** Schematic description of the natural history of breast cancer in three subsets of patients. Vertical axis: tumour volume (logarithmic scale) expressed by tumour diameter; horizontal axis: time. Zero time is the time at which the breast primary tumour, assumed to be spherical, has reached a clinically detectable size (tumour volume = 1 ml). Vertical arrows indicate the median size of the tumour at the time of the first, second, etc., axillary lymph node involvement. Horizontal arrows (M) indicate the median size of the tumour at the time of distant metastatic dissemination. Tumour growth pattern is assumed to be exponential in keeping with previous data. The three groups of patients are defined by the size $V_1$ of the tumour at the time of first axillary lymph node involvement. $\bar{V}_1$ is the median size for the whole group of operable patients. $\sigma$ is the standard deviation of $V_1$. Thus the $(\bar{V}_1-\sigma)$ group corresponds to patients with early first lymph node involvement and $(\bar{V}_1+\sigma)$ to that of late involvement. The tumour doubling times for the three groups were calculated according to the method described in the text.

the two lymphatic areas, since its value for internal mammary node involvement is 0.225.

Table III shows that among patients with tumours located in the inner quadrants, there is a much higher propensity for axillary node involvement in patients with invaded internal mammary nodes than in patients without internal mammary invasion. Thus there is a highly significant correlation between the propensity for axillary and internal mammary node invasions.

Among the 20 subsets of tumours defined by the five classes of tumour volumes and the four classes of the number of involved axillary lymph nodes, there is a strong

**Table II** Mean tumour volume at initiation of first distant metastases ($V_{50}$)

| | *All tumours* | *Outer tumours* | *Inner tumours* |
|---|---|---|---|
| | 12.32 ml | 13.82 ml | 9.17 ml |
| 95% confidence interval of the mean | 9.05–16.8 ml | 9.6–19.9 ml | 5.94–14.2 ml |

**Table III** Among patients with tumour located in the inner quadrants, proportion of patients with a given number of invaded axillary nodes according to the internal mammary chain status

| | *Percentage of patients with invaded axillary nodes* | | | |
|---|---|---|---|---|
| | *0* | *1–3* | *4–7* | *≥7* |
| Patients without internal mammary nodes involvement | 31.7% | 35.9% | 18.3% | 14.1% |
| | | | 68.3% | |
| Patients with internal mammary nodes involvement | 8.8% | 30.4% | 26.3% | 34.5% |
| | | | 91.2% | |

correlation between the mean volumes of the tumours at initiation of the first axillary node and of the first distant metastasis. The weighted correlation coefficient is equal to 0.934. Figure 5 displays the results which correspond to the following regression line:

$$\log V_m = a \log V_1 + b.$$

In patients with outer tumours, the best fit is obtained with $a = 0.479$ and $b = 2.85$. In patients with inner tumours, the best fit is obtained with $a = 0.51$ and $b = 2.32$. However, the values of $a$ in inner or outer tumours are not significantly different whereas the values of $b$ differ significantly.

In this relationship between $\log V_m$ and $\log V_1$, the slope $a$ provides an estimate of the correlation ($R_{V_1V_m} = R$) between the value $V_1$ and $V_m$ in a given patient. The relationship between the correlation and the slope $a$ is:

$$R = a(\sigma \log V_1 / \sigma \log V_m) = 0.5(4.555/4.836) = 0.47.$$

**Figure 5** Relationship between the breast tumour volume at metastatic dissemination and the breast tumour volume at the time of involvement of the first axillary lymph node. The volumes were calculated according to the method described in the text for 20 subgroups of patients defined by the tumour size at initial treatment and the number of involved axillary nodes (see text). However, it is noteworthy that there are, in the subsets with a small tumour volume, only small numbers of patients at involvement of first lymph node. This may explain the scatter for these subsets.

Thus for a given value of $V_1$ the residual variance of $V_m$ is equal to:

$$\sigma^2 \log V_m / \log V_1 = \sigma^2 \log V_m (1 - R^2) = 0.78\sigma^2 \log V_m.$$

Hence among patients with a given value of $\log V_1$ the variance of $\log V_m$ is reduced by 22% (Figure 6).

Whatever the location of the tumour in the inner or the outer quadrants, the variance of $\log V_m$ is identical. This would suggest that the correlation between the propensities for axillary lymph node involvement and for distant metastases are the same for inner and outer tumours. The significant difference observed for the value of the parameter $b$ between inner and outer tumours is probably related to the later involvement of the axillary nodes in tumours located in the inner quadrants.

Whatever the location of the tumour, when the volume of the tumour at initiation of the first node is increased 100-fold, the volume of the tumour at initiation of the first distant metastasis is increased approximately 10-fold (Figures 4 and 5). Both events are very rare: for example on average the tumour volume increases approximately 9-fold from the time at which the first axillary lymph node is involved to the time at which a second node is involved.

**Figure 6** Distribution (density of probability) of the tumour volume at metastatic dissemination for the whole patient population and for a patient population having the same tumour volume at initiation of the first axillary lymph node.

**Table IV** Proportion of patients (observed and calculated) with distant metastases according to the diameter of the breast tumour and the number of involved axillary nodes

| Axillary nodes | All patients Obs. | All patients Calc. | Tumour diameter 1.25 cm Obs. | Tumour diameter 1.25 cm Calc. | Tumour diameter 2.25 cm Obs. | Tumour diameter 2.25 cm Calc. |
|---|---|---|---|---|---|---|
| 0 | 0.251 | 0.261 | 18.6 ± 4.5 | 20.8 ± 0.2 | 23.3 ± 5.8 | 27.8 ± 0.3 |
| 1–3 | 0.461 | 0.461 | 37.6 ± 7.9 | 39.1 ± 0.3 | 48.0 ± 8.8 | 45.7 ± 0.3 |
| 4–7 | 0.582 | 0.593 | 42.7 ± 13.5 | 51.8 ± 0.4 | 52.8 ± 10.8 | 57.4 ± 0.4 |
| ⩾8 | 0.738 | 0.705 | 71.6 ± 15.6 | 60.8 ± 0.6 | 71.4 ± 10.7 | 68.2 ± 0.5 |

The proportion of patients with distant metastases can be estimated according to the number of involved axillary nodes and the tumour volume. As an example, Table IV compares in the overall population 2 subsets of patients, the first one with tumour diameter less than 2 cm (mean 1.25 cm) and the second with a tumour diameter between 2 and 3 cm (mean 2.25 cm). The overall correlation between observed and calculated values is highly significant.

### Discussion

The natural history of human breast cancers is a topic which has been much discussed but for which quantitative data has been scanty until the present studies (Tubiana & Koscielny, 1987; Koscielny *et al.*, 1984, 1985). The results presented in this paper quantify the concept of an orderly involvement of axillary nodes during tumour growth. Despite the wide spread of tumour volume at the invasion of the first axillary lymph node, the ratio of the tumour volume at invasion of the first and the second or the third lymph node is identical in all subgroups of patients. Thus the progressive involvement of axillary nodes follows the same probabilistic law in all patient subsets.

Another striking result is the demonstration of a strong correlation between the propensity for lymphatic involvement and the probability of distant haematogenous dissemination. It has been possible to characterise the propensity for lymph node invasion by the volume $V_1$ of the tumour at first node involvement. This volume was computed and found to be significantly correlated with the tumour volume ($V_m$) at metastatic spread in all subsets of patients (Figure 5). From a fundamental point of view, this is an important and an original result. It is consistent with recent findings showing the independent prognostic impact of tumour volume and number of positive nodes (Koscielny *et al.*, 1984; Atkinson *et al.*, 1986) and provides a basis for their interpretation.

The present results show that, on average, during tumour progression the capacity for lymphatic spread is acquired much earlier than the capacity for haematogenous spread. The acquisition of the capacity for lymphatic spread appears to be an event which occurs more frequently than the acquisition of the capacity for haematogenous spread.

It should be pointed out that the delay between the migration of the neoplastic cell which originates a colony in the axillary lymph nodes and that which originates distant metastases is even longer than depicted in Figure 4 and in Tables I and II. The method for the determination of the tumour size at initiation of first metastasis ($V_m$) estimates its size at the time of the seeding of the neoplastic cell. Conversely, a tumour cell colony in a lymph node becomes detectable when the number of cells in the colony reaches $10^3$ to $10^5$, so the seeding of the first neoplastic cells occurred at least 10 doubling times earlier. However, this methodological bias has no impact on the relative sizes of the tumours at the time of the first, second, third, etc., lymph node involvement in the various subgroups of patients.

Three groups of tumours are compared in Figure 4: average tumours ($V_1 = \bar{V}_1$), tumours with a high propensity to lymphatic spread ($V_1 = \bar{V}_1 - \sigma$), and tumours with a low propensity ($V_1 = \bar{V}_1 + \sigma$). The volume of the primary tumour at first nodal involvement is 100-fold larger in the median subset than in that with a high propensity and 100-fold smaller than in the subset with a low propensity. The differences in the volume at initiation of distant spread are 10 times smaller. These considerable differences are consistent with the concept of biological predetermination of cancer progression. Moreover these data (Figures 4 and 7) show that $V_1$ is a useful variable for quantifying this predetermination. The present analysis is not, however, consistent with the concept that breast cancer is a conglomerate of different diseases with widely different natural histories. It rather suggests that there is a continuum from slow growing disease with late axillary involvement and distant dissemination to the most aggressive, rapidly growing subtype.

Even in the worst prognostic subset, lymphatic and haematogenous spread are extremely rare events. For example, assuming a cell loss factor of 75% in a tumour belonging to the intermediate subset, then out of $2 \times 10^{10}$ cells which were born in the tumour, only one cell will migrate to a node and initiate the first nodal neoplastic colony. Moreover the birth of more than $10^{11}$ cells will occur before another cell will initiate the second neoplastic colony in another node. For haematogenous spread, the first colony is initiated after $10^{12}$ mitoses in the primary tumour.

As a consequence of this study, the prognostic significance of axillary lymph node involvement can be better interpreted and assessed. Intuitively, clinicians know that the prognostic significance of a given number of involved lymph nodes is not the same for small and large tumours. Fisher *et al.* (1970) and Handley (1972) reported that an increasing size of the primary tumour is usually correlated with an increasing likelihood of node invasion. However, these observations remained qualitative. The present data help one to

**Figure 7** Distribution (density of probability) of the tumour volume (in ml) at the time of distant metastatic dissemination in two of the three subsets of patients defined in Figure 3. The mean volume of the primary tumour at dissemination differs very significantly in the two groups, but there is in the three groups a wide spread of individual values and therefore a considerable overlapping of the three distributions.

understand some apparently paradoxical observations reported on breast cancers.

For tumour diameters ranging from 0.1 to 2 cm, the predictions of the model could be compared with the few available data (Bedwani *et al.*, 1981; De Metz *et al.*, 1987; Tabar *et al.*, 1987). However, the validity of this comparison is debatable. Some detectable tumours were not operated, for example because they were associated with palpable axillary nodes, or fixation to the thoracic wall or inflammatory reaction. These non-operable tumours are not included in this study, which comprises only tumours treated by surgery. Conversely, tumours detected by screening are all operated upon. The biological characteristics of tumours which remain operable when they reach a detectable size may differ from that of the non-operable tumours of the same size.

In a previous study, we showed that, in a population of patients, the probability of dissemination, expressed by the percentage of patients with distant spread, increases as a function of the tumour volume (Koscielny *et al.*, 1984). This increase can be interpreted by the growing proportion of tumours that are larger than their threshold volume $V_m$ at which the first distant metastasis is initiated. The distribution of $V_m$ values is log normal and the wide spread of the individual values corresponds to a large variance of the distribution (Figure 6). This distribution can be characterised by two parameters, the median ($V_{50}$) and the variance. Previous data showed that histological grade and number of involved lymph nodes markedly influence the $V_{50}$. However, the influence of these two variables on the variance of the $V_m$ distribution is relatively small. The present data show that the variance is reduced by about 20% when the size at the involvement of the first lymph node is taken into account (Figure 6). Thus this parameter is more informative than the number of involved lymph nodes but is still associated with only one-quarter of the variance. Therefore the search for other parameters associated with this large variance should continue.

The comparison between tumours located in the inner and outer quadrants of the breast shows that involvement of an axillary node is not a frequent step for metastatic spread since the volume at distant metastatic dissemination is the same for the two subsets of tumours, whereas the volume of the tumour at first axillary involvement is smaller for outer tumours then for inner tumours. This observation does not favour the so-called cascade model (Viadana *et al.*, 1978) and the traditional view of distant spread which assumes that the lymphatic system is the main pathway (Robbins & Cotran, 1979). Indeed, the data strongly suggest that the liability, for either lymphatic or haematogenous spread, although related, are nevertheless not linked by a causal relationship. Their correlation is probably due to the fact that both are caused by the characteristics of the neoplastic cells, in particular their genetic instability (Tubiana, 1986). The concept of tumour–host relationship does not appear to be required for the interpretation of the data.

This conclusion, namely that axillary involvement is a good index for the propensity of tumour cells in acquiring the capacity of haematogenous spread but not the cause of this spread, is consistent with the concept developed by Fisher (1984). Furthermore it is noteworthy that loco-regional recurrence rates are also much higher in patients with axillary involvement and are related to the number of invaded nodes (Devitt, 1967; Tubiana & Sarrazin, 1987). Hence lymphatic spread is also a signpost of the migration of tumour cells into the surrounding tissues. The study of oncogenes and of the biology of the tumour cells should help us to understand better the underlying molecular mechanisms.

The probabilities of axillary invasion and of internal mammary involvement are also highly correlated (Table III), which is in keeping with previous studies (Lacour *et al.*, 1976). Among patients with inner tumours, the data show that only 9% of the patients with an invaded internal mammary chain are without axillary involvement, as compared to 32% without axillary involvement when the internal mammary chain is not involved. Among operable patients with tumours of the inner quadrants and with involved internal mammary, the model predicts that approximately 45% of them have no distant metastases. This result is consistent with the data concerning our patient population and is in agreement with the effectiveness of the treatment of the internal mammary that we have recently emphasised (Lacour *et al.*, 1976; Tubiana *et al.*, 1986; Arriagada *et al.*, 1988).

Finally, one of the interesting features of the model is the inclusion into the evaluation of the probability of distant metastases of other factors besides $V_1$, such as the labelling index, histological grade, oestrogen receptors, etc. Some are highly correlated with $V_1$, and so will probably add little to the accuracy with which the probability of spread is presently determined. However, others, such as labelling index or tumour growth rate (Tubiana & Koscielny, 1988), have independent prognostic significance, so one might expect that they could improve the validity of the predictions. This is a point that we shall discuss further in a subsequent paper.

### Appendix

*Precision of the estimation of $V_1$*

Simulations have been performed in order to estimate the precision of the calculated volume at the first node involvement ($V_1$) in groups of patients defined by tumour volume at treatment and number of involved axillary lymph nodes (see Methods).

These simulations can be described as follows: for each tumour, the tumour volume at treatment and at the first lymph node invasion are taken at random, according to the observed distributions for these variables. These two volumes are taken as independent. The tumour volume at the time of the first metastasis initiation is calculated according to the volume at the first node invasion, assuming a positive correlation $(R_{v1,vm})=0.5$ (as found in the results) between these two volumes. These values of $V_1$ are termed, below, the exact ones.

The volumes at the successive lymph nodes invasions are thereafter calculated as a function of the volume at the first lymph node invasion. The volume at node $n$ is equal to the volume at node $n-1$ multiplied by a factor $k_n$. The value $k_n$ is defined by the data for the relationships between tumour size and the number of involved axillary lymph nodes. The values of $k_n$ can be supposed either the same for the tumours or varying from one tumour to another. If $k_n$ is assumed variable, each $k_n$ value is taken at random from a uniform distribution with an interval $0-2\mu(k_n)$. For example, $k_n$ equal to 0 means that initiation of nodes $n$ and $n+1$ occurs at the same time.

The tumour volumes at the time of successive node involvements and the volume at treatment are calculated. The number of involved axillary nodes at the time of tumour treatment is obtained by comparing the volume at treatment to the volumes at the different node invasions. The 'apparent' tumour volume at the first node involvement is therefore calculated with the procedures described in the article, according to the volume at treatment and the number of involved nodes.

This procedure is iterated for 2,408 tumours (the size of our population). In order to calculate the values of the means of the exact values and of the apparent values. The parameters of the distribution of the mean value of exact $V_1$ and of apparent $V_1$ are estimated from the simulation of 1,000 populations.

The difference between the apparent mean volume at the initiation of the first lymph node and the exact value is less than 0.1%. The variance of the estimation of the mean is less than the variance of the exact mean, especially in the subgroups with no involved lymph node.

The estimation of the parameters of the relationship between the volume at the initiation of the metastases and at the initiation of the first lymph node is not affected by the variability on the ratio $k_n$. A possible bias in the analysis can be caused by variation of the variance of the tumour volume at the initiation of the first metastasis ($V_m$) in the various subgroups of tumours. This variance is assumed to be the same in the subgroups as in the whole population. However, as $V_1$ and $V_m$ are correlated, the variance of $V_m$ depends on the variance of $V_1$. The variance of the tumour volume at metastasis initiation is almost the same in the various groups and nearly equal to the variance estimated for the whole population. The variance of the tumour volume at the first lymph node initiation is relatively large in each group.

In fact, the number of involved nodes only gives information concerning the range of volume values including $V_1$. Consequently, the variance of $V_1$ estimation is relatively large. Moreover, the variance of the volume at the first metastasis initiation in the different groups only depends, in this context, on the variance of $V_1$ in the corresponding group.

$$\sigma^2 V_m/V_1 = \sigma^2 V_m[1 - R^2 V_1 V_m(1 - \sigma^2 V_{1g}/\sigma^2 V_1)]$$

where $\sigma^2 V_{1g}$ is the variance of $V_1$ in the subgroup. As $\sigma^2 V_{1g}$ is very close to $\sigma^2 V_1$, the value of $\sigma^2 V_m/V_1$ in the various groups can be considered as equal to the value of $\sigma^2 V_m$ estimated for the whole population.

We wish to express our sincere thanks to all the physicians who are involved at Institut Gustave-Roussy in the management of patients with breast cancer, in particular Dr G. Contesso (Pathology), Dr J.C. Delarue (Clinical biology), Dr D. Castaigne, Dr P. Lasser, Dr J.Y. Petit, Dr F. Rochard, Dr J.P. Travagli (Surgery), Dr T. Le Chevalier, Dr F. May-Levin, Dr J. Rouesse, Dr M. Spielmann (Medical Oncology), Dr R. Arriagada, Dr F. Fontaine, Dr D. Sarrazin (Radiotherapy), Mrs H. Mouriesse (Medical Statistics). This work would have been impossible without the care with which they have kept the breast cancer registry of the Institut Gustave-Roussy. Mr Andrew Kramar from the department of biostatistics and epidemiology and Mr N. Blackett carefully read the manuscript. Their remarks and criticisms were most helpful.

### References

ARRIAGADA, R., LE, M.G., MOURIESSE, H. and 7 others (1988). Long-term effect of internal mammary chain treatment. Results of a multivariate analysis of 1,195 patients with operable breast cancer and positive axillary nodes. *Radiother. Oncol.*, **11**, 213.

ATKINSON, E.N., BROWN, B.W. & MONTAGUE, E.D. (1986). Tumor volume, nodal status and metastasis in breast cancer women. *J. Natl Cancer Inst.*, **76**, 171.

BEDWANI, R., VANA, J., ROSNER, D., SCHMITZ, R.L. & MURPHY, G.P. (1981). Management and survival of female patients with 'minimal' breast cancer: as observed in the long-term and short-term surveys of the American College of Surgeons. *Cancer*, **47**, 2769.

BRINKLEY, D. & HAYBITTLE, J.L. (1977). The curability of breast cancer. *World J. Surg.*, **1**, 287.

CONTESSO, G., ROUESSE, J., PETIT, J.Y. & MOURIESSE, H. (1977). Les facteurs anatomo-pathologiques du pronostic des cancers du sein. *Bull. Cancer (Paris)*, **64**, 525.

DE METZ, C.E. & PORTER, A.T. (1987). The significance of tumor size in stage I invasive breast cancer. In *Fundamental Problems in Breast Cancer*, Paterson, A.H.G. & Lees, A.W. (eds) p. 91. Martinus Nijhoff: Boston.

DEVITT, J.E. (1967). The clinical stages of breast cancer – what do they mean? *Can. Med. Assoc. J.*, **97**, 1257.

DRAPER, N.K. & SMITH, H. (1981). *Applied Regression Analysis*, 2nd ed. Wiley: New York.

FINNEY, D.J. (1964). *Statistical Method in Biological Assay*, 2nd. ed. Griffin: London.

FISHER, B. & SLACK, N.H. (1970). Number of lymph nodes examined and the prognosis of breast cancer. *Surg. Gynecol. Obstetr.*, **131**, 79.

FISHER, B. (1984). The role of science in the evolution of breast cancer management. Health Memorial Award Lecture. In *Current Controversies in Breast Cancer*, Ames, F.C., Blumenschein, G.R. & Montague, E.D. (eds) p. 1. University of Texas Press: Austin.

FISHER, E.R., SASS, E. & FISHER, B. (1984). Pathological findings from the NSABP Protocol 4: discriminants for tenth year treatment failure. *Cancer*, **53**, 712.

HANDLEY, R.S. (1972). Observations and thoughts on cancer of the breast. *Proc. R. Soc. Med.*, **65**, 437.

KOSCIELNY, S., TUBIANA, M., LE, M.G. and 4 others (1984). Breast cancer, relationship between the size of the primary tumour and the probability of metastatic dissemination. *Br. J. Cancer*, **49**, 709.

KOSCIELNY, S., TUBIANA, M. & VALLERON, A.J. (1985). A simulation model of the natural history of human breast cancer. *Br. J. Cancer*, **52**, 515.

LACOUR, J., BUCALOSSI, P., CACERES, E. and 5 others (1976). Radical mastectomy versus radical mastectomy plus internal mammary dissection. Five-year results of an international co-operative trial in breast cancer. *Cancer*, **37**, 206.

LACOUR, J., JURET, P. & SARRAZIN, D. (1968). Protocole schématique de traitement des cancers du sein à l'Institut Gustave-Roussy. *Rev. Prat. (Paris)*, **18**, 3595.

ROBBINS, S.L. & COTRAN, R.S. (1979). Neoplasia. In *Pathologic Basis of Diseases*, 2nd edn., p. 156. W.B. Saunders: Philadelphia.

SARRAZIN, D., LE, M., MOURIESSE, H. and 4 others (1982). Radiotherapeutic studies on breast cancer at Villejuif. *Cancer Bull. (Houston)*, **34**, 242.

TABAR, L., FABERBERG, G., DAY, N.E. & HOLMBERG, L. (1987). What is the optimum interval between mammographic screening examinations? An analysis based on the latest results of the Swedish two-country breast cancer screening trial. *Br. J. Cancer*, **55**, 547.

TUBIANA, M. (1986). The growth and progression of human tumors: implication for management strategy. *Radiother. Oncol.*, **6**, 167.

TUBIANA, M., ARRIAGADA, R. & SARRAZIN, D. (1986). Human cancer natural history, radiation induced immunodepression and postoperative radiation therapy. *Int. J. Radiat. Oncol. Biol. Phys.*, **12**, 477.

TUBIANA, M. & KOSCIELNY, S. (1987). The natural history of human breast cancer: implications for patient management. In *Fundamental Problems in Breast Cancer*, Paterson, A.H.G. & Lees, A.W. (eds) p. 333. Martinus Nijhoff: Boston.

TUBIANA, M. & KOSCIELNY, S. (1988). Cell kinetics, growth rate and the natural history of breast cancer. *Eur. J. Cancer Clin. Oncol.*, **24**, 9.

TUBIANA, M. & SARRAZIN, D. (1987). The role of post-operative radiotherapy in breast cancer. In *Breast Cancer: Diagnosis and Treatment*, Ariel, J.M. & Cleary, J.B. (eds) p. 280. McGraw-Hill: New York.

VIADANA, E., BROSS, I.D. & PICKREN, J.W. (1978). Cascade spread of blood-borne metastases in solid cancers of humans. In *Pulmonary Metastasis*, Weiss, L. & Gilbert, H.A. (eds) p. 142. G.K. Hall: Boston.